# Validation of a 5-Item Tool to Measure Patient Assessment of Clinician Compassion in Hospitals

**DOI:** 10.1007/s11606-021-06733-5

**Published:** 2021-04-09

**Authors:** Brian W. Roberts, Michael B. Roberts, Anthony Mazzarelli, Stephen Trzeciak

**Affiliations:** 1 Department of Emergency Medicine, Cooper University Health Care, Cooper Medical School of Rowan University, Camden, NJ USA; 2 Center for Humanism, Cooper Medical School of Rowan University, Camden, NJ USA; 3 Cooper University Hospital, Camden, NJ USA; 4 Institutional Research and Outcomes Assessment, Philadelphia College of Osteopathic Medicine, Philadelphia, PA USA; 5 Department of Medicine, Cooper University Health Care, Cooper Medical School of Rowan University, Camden, NJ USA

**Keywords:** compassion, empathy, patient satisfaction, patient experience

## Abstract

**Background:**

We previously validated a 5-item compassion measure to assess patient experience of clinician compassion in the outpatient setting. However, currently, there is no validated and feasible method for health care systems to measure patient experience of clinician compassion in the inpatient setting across multiple hospitals.

**Objective:**

To test if the 5-item compassion measure can validly and distinctly measure patient assessment of physician and nurse compassion in the inpatient setting.

**Design:**

Cross-sectional study between July 1 and July 31, 2020, in a US health care network of 91 community hospitals across 16 states consisting of approximately 15,000 beds.

**Patients:**

Adult patients who had an inpatient hospital stay and completed the Hospital Consumer Assessment of Healthcare Providers and Systems (HCAHPS) survey.

**Measurements:**

We adapted the original 5-item compassion measure to be specific for physicians, as well as for nurses. We disseminated both measures with the HCAHPS survey and used confirmatory factor analysis for validity testing. We tested reliability using Cronbach’s alpha, as well as convergent validity with patient assessment of physician and nursing communication and overall hospital rating questions from HCAHPS.

**Results:**

We analyzed 4756 patient responses. Confirmatory factor analysis found good fit for two distinct constructs (i.e., physician and nurse compassion). Both measures demonstrated good internal consistency (alpha > 0.90) and good convergent validity but reflected a construct (compassionate care) distinct from what is currently captured in HCAHPS.

**Conclusion:**

We validated two 5-item tools that can distinctly measure patient experience of physician and nurse compassion for use in the inpatient hospital setting in conjunction with HCAHPS.

**Supplementary Information:**

The online version contains supplementary material available at 10.1007/s11606-021-06733-5.

## INTRODUCTION

The construct of compassion is commonly defined as the emotional response to another’s pain or suffering involving an authentic desire to help.^1–3^ Compassion is differentiated from empathy in that empathy is defined as an affective state of sensing and understanding another’s emotions. In other words, in healthcare, empathy is a feeling the clinician has, as opposed to compassion, which encompasses positive behaviors which patients can perceive.^3^ Clinician compassion during patient care is considered by both patients and physicians to be a vital element of high-quality care,^4^ and previous research has shown compassion to be associated with better clinical outcomes across numerous conditions.^5–10^ In addition, focus on compassionate care may have financial impact on healthcare organizations given that a lack of compassion has been associated with increased resource utilization, healthcare spending, and malpractice expense.^11–17^

Given that patient perception of compassionate care (as opposed to self-assessment of compassion by the clinician or third party observer assessment of compassion) is associated with patient outcomes and healthcare costs, we previously developed and validated a 5-item compassion measure to assess patient experience of clinician compassion in the outpatient setting,^18^ which can be administered with the Clinician and Group Consumer Assessment of Healthcare Providers and Systems (CG-CAHPS) survey. The CG-CAHPS is a patient satisfaction survey for adult outpatient clinic visits used by the United States (US) Centers for Medicare and Medicaid Services for all healthcare organizations that receive payments from Medicare.^19^ While we initially validated the 5-item compassion measure in the outpatient setting, assessing patients’ perception of compassion from a single provider, we subsequently tested the validity of the 5-item compassion measure to assess patients’ overall perception of compassion (i.e., perceived aggregate compassion from multiple clinicians) across three US emergency departments (ED) and found it to be reliable and valid in this setting as well.^20^ It is further possible that the 5-item compassion measure could be utilized to assess overall clinician compassion during hospitalization by administering it with the CAHPS Hospital Survey (HCAHPS), similar to HCAHPS methods in assessing overall patient experience. The 5-item compassion measure has also not been previously validated to differentiate between different types of clinicians (e.g., physicians and nurses) as the HCAHPS survey has. There is currently evidence that compassion in healthcare is suboptimal worldwide.^5^ This is a significant public health issue given the impact that compassion (or lack thereof) can have on outcomes for patients and healthcare organizations. Thus, having the means to measure patient assessment of compassionate care in hospitals on a large scale is important for healthcare quality.

The objectives of this study were to (1) psychometrically validate the 5-item compassion measure when administered with the HCAHPS survey for inpatient hospital care, and (2) to test if the 5-item compassion measure is a valid and reliable tool to quantify two distinct constructs (i.e., physician compassion and nurse compassion) for hospitalized patients.

## METHODS

### Setting

This study was conducted across a US acute hospital system, which consists of 91 hospitals within 16 states (Alabama, Alaska, Arizona, Arkansas, Florida, Georgia, Indiana, Mississippi, Missouri, New Mexico, North Carolina, Oklahoma, Pennsylvania, Tennessee, Texas, West Virginia). The included institutions are community hospitals without any associations to universities and compose approximately 15,000 beds in total. The study took place from July 1 to July 31, 2020. Given this research involved only survey procedures and information was recorded in such a manner that subjects could not be identified, the Institution Review Board at our institution (Cooper University Health Care, Camden, NJ) considered this study exempt from the 45 Code of Federal Regulations (CFR) requirements as per regulation 45 CFR 46.101(b).^2, 18, 21^ This study is reported in accordance with the Strengthening the Reporting of Observational Studies in Epidemiology (STROBE) Statement.^22^

### Study Population

The study population included patients’ age ≥ 18 who had at least one overnight stay in the hospital as an inpatient and completed the HCAHPS survey. We matched our inclusion criteria to correspond with the HCAHPS survey given that a primary objective of this measurement tool is to work in conjunction with the HCAHPS platform for dissemination at scale (see Supplemental Methods).

### 5-Item Compassion Measure

For the purposes of this study, and for dissemination with the HCAHPS survey, each of the five items of the original measurement tool were modified to specifically elicit separate responses for interactions with physicians and nurses (i.e., 10 items total) and are displayed in Table 1 (see Supplemental Methods).

**Table 1:** Compassion Measure Items for Both Physicians and Nurses. Each Item Response Scaled as 1=Never; 2=Sometimes; 3=Usually; 4=Always

Items	
Physicians	
During this hospital stay, how often do you feel your doctors cared about your emotional or psychological well-being?	
During this hospital stay, how often do you feel your doctors were interested in you as a whole person?	
During this hospital stay, how often do you feel your doctors were considerate of your personal needs?	
During this hospital stay, how often do you feel your doctors were able to gain your trust?	
During this hospital stay, how often do you feel your doctors showed you care and compassion?	
Nurses	
During this hospital stay, how often do you feel your nurses cared about your emotional or psychological well-being?	
During this hospital stay, how often do you feel your nurses were interested in you as a whole person?	
During this hospital stay, how often do you feel your nurses were considerate of your personal needs?	
During this hospital stay, how often do you feel your nurses were able to gain your trust?	
During this hospital stay, how often do you feel your nurses showed you care and compassion?	

### Statistical Analysis

Patient survey responses were described using median and interquartile range (IQR) for continuous variables and frequency and proportions for categorical variables.

Separate confirmatory factor analyses (using structural equation modeling) were used to test how well the 5-item compassion measure assessed a single construct of compassion from physicians and a separate single construct for compassion from nurses, as well as to calculate standardized coefficients for each item. Structural equation modeling tests if the hypothesized model (i.e., two discrete constructs, compassion from physicians, and compassion from nurses) matches the observed data. The Supplemental Methods further describe these models. Reliability was tested separately for the physician and nurse 5-item compassion measures, using Cronbach’s alpha. Cronbach’s alpha is a measure of internal reliability, or how closely the set of items in a measurement tool are related as a group.

We summed the scores for each individual item to obtain two separate composite scores, one for the physician 5-item compassion measure and one for the nurse 5-item compassion measure. Using Spearman correlation coefficients, we tested divergent validity between the two 5-item compassion measure total scores. We hypothesized that while the two 5-item compassion measures would have a positive correlation, they would be distinct from one another. To further test if the items from the two 5-item compassion measures form discrete constructs (i.e., measure physician and nursing compassion separately as opposed to a single construct of overall compassion), we used structural equation modeling to test model fit for a single construct model (combining physician and nurse tools as a single measure) versus a two-construct model with physician and nurse compassion measured separately.^18^ The Supplemental Methods further describes these analyses.

Pairwise Spearman correlation coefficients were used to test convergent validity between the physician 5-item compassion measure total scores and the HCAHPS physician communication questions (ability to listen, explain, and show courtesy and respect) and overall hospital rating (i.e., HCAHPS question, using any number from 0 to 10, where 0 is the worst hospital possible and 10 is the best hospital possible, what number would you use to rate this hospital?). We repeated this procedure for the nurse 5-item compassion measure. We hypothesized that while the two 5-item compassion measures would have a positive correlation with the HCAHPS constructs (i.e., patients who report higher compassion would also report better communication and overall hospital rating), these correlations would not be perfect (i.e., would be distinct and not redundant measures).

We further evaluated convergent and divergent validity between the 5-item compassion measures and the above HCAHPS constructs using structural equation modeling to perform mediation (i.e., causal pathway) testing. In building our model, we first designated four latent variables: (1) physician compassion; (2) nursing compassion; (3) physician communication (HCAHPS physician communication questions); and (4) nursing communication (HCAHPS nursing communication questions). We tested the associations between these four latent variables and overall hospital rating obtained from the HCAHPS. We further tested to what degree physician communication mediated the association between physician compassion and overall hospital rating (i.e., hypothesized model: increased physician compassion leads to better communication, which in turn leads to improved overall hospital rating). We also tested if physician communication mediated the association between nursing compassion and overall hospital rating. We expected (1) all four latent variables to be independently associated with overall hospital rating (convergent validity), (2) physician communication to partially mediate the association between physician compassion and overall hospital rating (i.e., while physician compassion and communication are expected to overlap, full mediation would suggest physician communication and physician compassion are not distinct constructs), and (3) if patients are able to distinguish between physician and nurse constructs, then physician communication should be independent of nurse compassion and will not mediate the association between nursing compassion and overall hospital rating (divergent validity). We repeated the mediation testing using nurse communication as the mediator. To estimate the mediation models, we used full information maximum likelihood and used robust standard errors clustered at the hospital level.

## RESULTS

**Figure 1:**
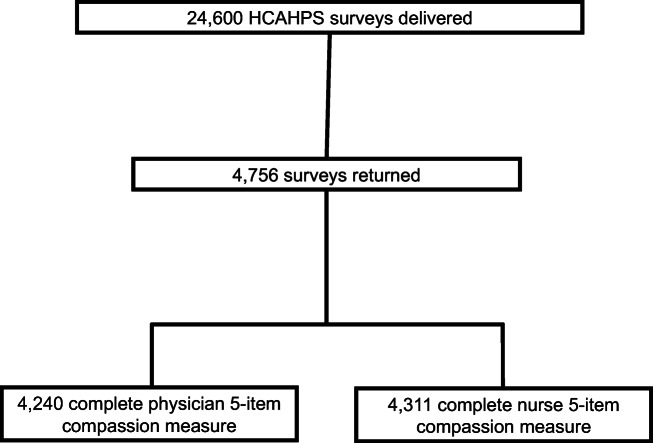
Study flow diagram.

Response rates are displayed in Figure 1. Of the 91 hospitals included, the median (IQR) number of responses included in the analyses per hospital was 41 (21-73). Patient self-reported characteristics are displayed in Table 2. The age range was 18 to 100 years old, 60% of participants were female, 57% had some degree of college education, and the majority were non-Hispanic white.

**Table 2:** Patient Characteristics

Variable	Cohort (*n* = 4756)
Age (mean (SD))	63 (18)
Female (*n* (%))	2867 (60)
Race (n (%))	
Non-Hispanic White	3799 (80)
Black/African American	334 (7)
Asian	51 (1)
Hawaiian/other Pacific Islander	16 (0.3)
American Indian/Alaska Native	66 (1)
Unknown	490 (10)
Hispanic or Latino decent (*n* (%))	272 (7)
Highest education level completed (*n* (%))	
8th grade or less	125 (3)
Some high school	299 (6)
High school graduate	1330 (28)
Some college	1454 (31)
4 years college graduate	616 (13)
4+ years college	628 (13)
Unknown	304 (6)
Self-reported overall health (*n* (%))	
Poor	239 (5)
Fair	887 (19)
Good	1552 (33)
Very good	1245 (26)
Excellent	635 (13)
Unknown	198 (4)

*SD*, standard deviation

Confirmatory factor analysis found all five items loaded well on separate single constructs for both the physician and the nurse measures (Table 3). We found both models had a good fit to the observed data (Supplemental Results). Internal reliability was excellent for both the physician and nursing 5-item compassion measures, Cronbach’s alpha = 0.96 and 0.95 respectively.

**Table 3:** Standardized Coefficients from Confirmatory Factor Analysis

	Standardized coefficient
Physician 5-item compassion measure	
Cared about your emotional or psychological well-being?	0.90
Was interested in you as a whole person?	0.91
Was considerate of your personal needs?	0.91
Was able to gain your trust?	0.90
Showed you care and compassion?	0.91
Nurse 5-item compassion measure	
Cared about your emotional or psychological well-being?	0.84
Was interested in you as a whole person?	0.90
Was considerate of your personal needs?	0.91
Was able to gain your trust?	0.89
Showed you care and compassion?	0.90

**Figure 2:**
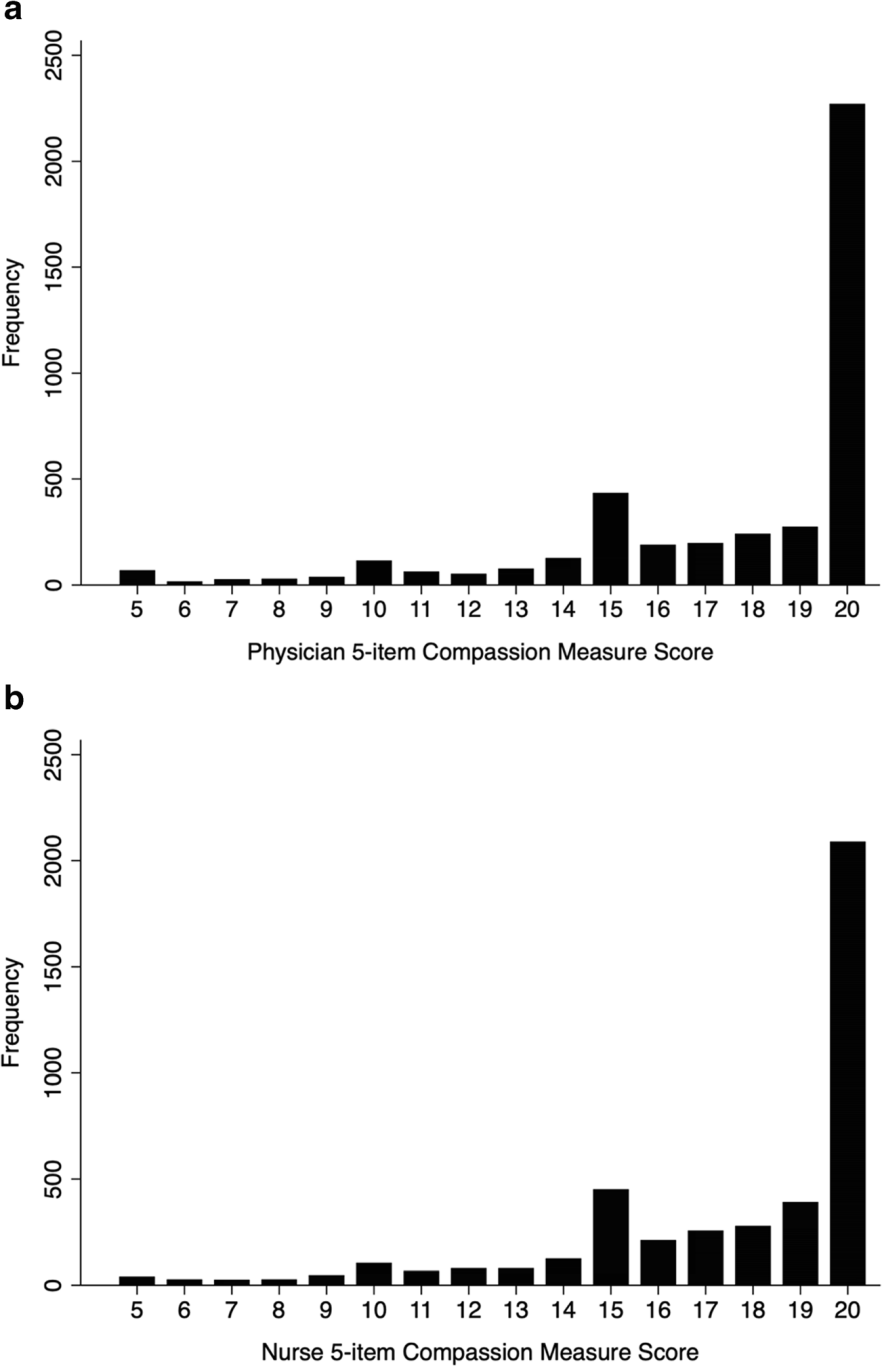
Frequency distribution of the **a** physician and **b** nurse 5-item compassion measure scores.

The 5-item compassion measure for both physicians and nurses ranged the full scale (5 to 20) Consistent with trends in patient satisfaction data in the USA, the responses trended favorably (i.e., negatively skewed).^18, 23^ The distributions for both the physician and nurse 5-item compassion measure scores are displayed in Figure 2.

The physician 5-item compassion measure had only a moderate correlation with the nurse 5-item compassion measure (i.e., while they trend in the same direction, they are discrete and not redundant) *r* = 0.68, *p* < 0.001. Using confirmatory factor analysis, we found a two-factor model (physician and nurse 5-item compassion measures loading on separate latent variables) to have a better fit to the observed data, compared to the single factor model (both the physician and nurse 5-item compassion measures loading on a single latent variable) (Supplemental Results). These results provide further evidence that the physician and nurse compassion measures are measuring discrete constructs.

We found the physician 5-item compassion measure to only have a moderate association with the HCAHPS physician communication and overall hospital rating, *r* = 0.69 (*p* < 0.001) and *r* = 0.55 (*p* < 0.001) respectively. Similar results were found for the nurse 5-item compassion measure and the HCAHPS nursing communication and overall hospital rating, *r* = 0.69 (*p* < 0.001) and *r* = 0.62 (*p* < 0.001) respectively. As we hypothesized, these results suggest the compassion measures trend in the same direction as the HCAHPS communication questions but do not simply reflect a redundant measure of patient experience already captured by the HCAHPS questions.

Our mediation model found all four latent variables to be independently associated with overall hospital rating (direct associations displayed in Table 4). As expected, physician compassion was associated with physician communication (*β* = 0.52 (95% CI 0.48 to 0.55)), and physician communication partially mediated the association between physician compassion and overall hospital rating (indirect effect, *β* = 0.27 (95% CI 0.12 to 0.42)). Thus, physician communication mediated only 44% of the total association between physician compassion and overall hospital rating. In other words, this model suggests physician compassion is a distinct construct that may improve physician communication, and further physician compassion may directly improve overall hospital rating, as well as have an indirect effect on overall hospital rating through its effects on physician communication.

**Table 4:** Regression Coefficients from the Structural Equation Model Testing the Direct Association between Physician Compassion, Nurse Compassion, Physician Communication, Nurse Communication (Independent Variables) and Overall Hospital Rating (Dependent Variable)

Variables	β coefficient	95% CI	*p* value
Physician compassion	0.35	0.17–0.52	< 0.001
Nurse compassion	0.43	0.21–0.64	< 0.001
Physician communication	0.53	0.24–0.82	< 0.001
Nurse communication	2.32	1.99–2.65	< 0.001

Furthermore, as expected, we did not find nurse compassion to be associated with physician communication (*β* = 0.02 (95% CI -0.01 to 0.05)), and physician communication did not mediate the association between nurse compassion and overall hospital rating (*β* = 0.01 (95% CI -0.003 to 0.03)). These results further suggest nurse compassion is a distinct construct that does not influence physician communication. Similar results were found using nurse communication (Supplemental Results).

## DISCUSSION

This study provides the initial validation of two measurement tools for patient assessment of physician and nurse compassion in hospitals. We found the two 5-item compassion measures were able to reliably assess physician and nurse compassion separately. Mediation testing found that while there were important associations between compassion, communication, and overall hospital rating, the physician and nurse 5-item compassion measures allow for specific assessments of compassion, which are distinct from, and add additional information to, the HCAHPS questions. For example, if we had found compassion was not associated with communication or overall hospital rating at all, it would have brought convergent validity into question. Alternatively, if communication mediated 100% of the association between compassion and overall hospital rating, it would have suggested that there was no difference between the constructs measured by the 5-item compassion measures and the constructs measured by the HCAHPS communication questions (i.e., redundant measures). Also, if physician compassion was found to be associated with nurse communication, or if nurse communication mediated the association between physician compassion and overall hospital rating, it would have suggested the measures were unable to distinguish separate physician and nurse constructs (i.e., our results support appropriate divergent validity). Thus, these models provide evidence that the physician and nurse 5-item compassion measures are able to measure distinct constructs.

The physician and nurse 5-item compassion measures are novel in that (1) they are patient assessments of compassion, as opposed to a third party observer or self-assessment (which is important because it is likely the patients’ experience that drives the association between compassion and clinical outcomes)^10, 18^; (2) they are brief, allowing dissemination with HCAHPS for large-scale assessment of compassion across healthcare organizations; and (3) they allow for distinct assessments of nurse and physician compassion, as opposed to an overall measure of clinician compassion.

The validation of the 5-item compassion measure in the inpatient setting has implications for future research. Being compassionate is not simply an inherent trait, which clinicians either do or do not possess, but evidence supports that compassionate behaviors can in fact be taught and learned.^2, 24^ The use of an easily distributed, validated measurement tool for patient assessment of compassion will allow for rigorous testing of interventions aimed at increasing clinician compassion and improving subsequent long-term outcomes (both clinical and economic).^18^ For example, by being able to measure compassion on a large scale across healthcare organizations, it may be possible to rapidly identify where within healthcare institutions compassionate care may be lacking, allowing for testing of focused interventions to increase compassion. In addition, the results of our mediation testing support patient assessment of compassion as a potential target to improve clinician-patient communication, as well as overall patient satisfaction. While our hypothesized causal pathway suggests increasing compassion may improve communication, it is similarly reasonable to hypothesize that improving communication may lead to greater perceived compassion. Future research is warranted to further evaluate these associations. We also believe future research is required to examine the impact of patient-clinician concordance/discordance in gender, race, and age on patient perception of compassionate care, as well as to validate the 5-item compassion measure in other languages.

We acknowledge that this study has important limitations to consider. First, the response rate in this study was consistent with low response rates seen in previous studies involving HCAHPS surveys.^18, 25, 26^ However, healthcare organizations can only estimate their patient experience metrics using data from responders, and the results of the psychometric analyses among those who did respond across these 91 hospitals suggest the physician and nurse 5-item compassion measures are valid and reliable assessments of patients’ perception of compassion. However, research is warranted to determine if non-response bias affects overall patient experience metrics. Similarly, we only tested the validity and reliability of the 5-item compassion measure on individuals who were sent the HCAHPS survey. Thus, these results cannot be generalized to those excluded from the HCAHPS survey. It is also important to note that the majority of the respondents were non-Hispanic white. Second, while the results of this study support that physician and nursing compassion can be measured distinctly, the study only supports the use of the 5-item compassion measure to obtain an overall physician and an overall nursing compassion score over the course of the entire hospitalization. While the 5-item compassion measure was previously demonstrated to be reliable and valid in assessing the compassion of a single physician in the outpatient setting,^18^ further research is required to test if the 5-item compassion measure can be used to assess individual physicians and nurses in the inpatient setting where patients typically are cared for by multiple clinicians. Specifically, research is required to determine how the compassion scores are affected when patients do not experience the same degree of compassion from all doctors and all nurses. Third, while we found patient assessment of compassion to be associated with clinician communication, further research is needed to determine what other clinician behaviors, as well as what non-clinician variables (e.g., severity of illness, hospital factors, timing of measurements) are associated with patient assessment of compassion. Interestingly, while we found a similar association between physician compassion and communication as we did for nurse compassion and communication, nurse communication had a stronger association with overall hospital rating and mediated more of the association between nurse compassion and overall hospital rating. This is likely secondary to nurse compassion and communication having a stronger total association with overall hospital rating compared to the physician constructs, possibly due to the fact that in general, nurses spend more time with the patient throughout the hospitalization. Fourth, this study did not assess advanced nurse practitioners or physician assistants. Fifth, this study was performed during the coronavirus disease 2019 (COVID-19) pandemic and the number of patients hospitalized for COVID-19 during our study period is unknown. However, we have no reason to suspect the pandemic would affect the validity or reliability of the 5-item compassion measure given our previous work performed outside of the pandemic.^18, 20^

In summary, the physician and nurse 5-item compassion measures appear to be reliable and valid tools to distinctly measure patient assessment of physician and nurse compassion in the inpatient setting across multiple hospitals. Further testing among varying cohorts is warranted to further test the generalizability of these measurement tools.

## Supplementary Information

ESM 1 (DOCX 33 kb)
