# Supplementary material for: Validation of a 5-Item Tool to Measure Patient Assessment of Clinician Compassion in Hospitals
Source: J Gen Intern Med. 2021 Apr 9;37(7):1697–703. doi: 10.1007/s11606-021-06733-5 (PMC8034051; doi:10.1007/s11606-021-06733-5)
Supplement: Supplementary file 1 — (DOCX 33 kb) [file 11606_2021_6733_MOESM1_ESM.docx]

**Validation of a 5-item tool to measure patient assessment of clinician compassion in hospitals**

Brian W. Roberts, MD, MSc^1,2^; Michael B. Roberts, PsyD^3^;

Anthony Mazzarelli, MD, JD, MBE^1,2^; Stephen Trzeciak, MD, MPH^2,4^

**Supplemental Material**

1: Department of Emergency Medicine, Cooper University Health Care, Cooper Medical School of Rowan University, Camden, NJ

2: Center for Humanism, Cooper Medical School of Rowan University, Camden, NJ

3: Institutional Research and Outcomes Assessment, Philadelphia College of Osteopathic Medicine, Philadelphia, PA

4: Department of Medicine, Cooper University Health Care, Cooper Medical School of Rowan University, Camden, NJ

For submission to *Journal of General Internal Medicine*

Address for correspondence:

Brian Roberts, MD, MSc

Cooper University Hospital

One Cooper Plaza, K152

Camden, New Jersey 08103

Phone: (856) 342-2351

E-mail: roberts-brian-w@cooperhealth.edu

Supplemental Methods

*Consumer Assessment of Healthcare Providers and Systems Hospital Survey (HCAHPS)*

The HCAHPS survey is a 29-item survey instrument and data collection methodology for measuring patients’ perceptions of their hospital experience and is the first national, standardized, publicly reported survey of patients' perspectives of hospital care, developed by the Centers for Medicare & Medicaid Services in conjunction with the Agency for Healthcare Research and Quality.(1) Patients are excluded from receiving a HCAHPS survey if they (1) have a principle psychiatric Medicare Severity-Diagnosis Related Group diagnosis at the time of hospital discharge; (2) do not have a valid U.S. mailing address or cell phone number; (3) are discharged to hospice care, nursing homes, or skilled nursing facilities; or (4) are a prisoner. At each hospital, data for patients discharged five days prior are sent daily to the survey vendor (Press Ganey Associates, Inc.). The vendor sends out surveys to patients within 48 hours of receiving the data. The vendor collates responses and returns the deidentified survey data to the health system.

*5-item compassion measure*

The 5-item compassion measure is a simple, parsimonious tool used to measure the construct of clinician compassion, defined as an emotional response to another’s pain or suffering involving an authentic desire to help,(2-5) from the patient perspective. The 5-item compassion measure was prospectively developed and validated in the outpatient setting across 15 different specialties,(5) as well as validated for use in the emergency department.(6)

*Confirmatory factor analyses*

Confirmatory factor analyses (using structural equation modeling) were used to evaluate how correctly the hypothesized models matched the observed data. Given the non-normality of the data (i.e. ordinal data) we used the Satorra and Bentler scaled chi-square test, which provides a scaled version of the chi-square statistic that more closely follows the mean of the reference distribution in the presence of non-normally distributed data.(7) As previously performed, we examined fit indices (which take into account total sample size), including Comparative Fit Index (CFI), Tucker-Lewis Index (TLI), and standardized root mean squared residual (SRMR). *A priori*, we chose our model to have good fit if CFI > 0.95, TLI > 0.95, and SRMR < 0.08.(5, 6, 8) We chose to examine fit indices because, when the sample size is large, the chi-square test for model fit is often significant (i.e. suggesting the hypothesized model is a poor fit) even when the model is, in practice, a good fit (i.e. large sample sizes will result in rejection of the hypothesized model based on insignificant deviations from the observed data).(5, 6, 9, 10)

*Nested models testing single construct versus two construct model*

Using structural equation modeling we tested the null hypothesis that the covariance between the two latent structures (physician and nurse compassion) is 1 (i.e. single construct model).(5) To test this hypothesis we used a likelihood ratio test to compare two nested models: one model with covariance between the two latent models constrained at 1 (i.e. single construct) versus a second model with covariance between the two latent models allowed to be a free parameter (i.e. two construct model with physician and nurse compassion measured separately).(5) We also report the fit indices for the two-construct model and used the *a priori* definition for good fit described above.

Supplemental Results

*Physician and nurse 5-item compassion measure model fit*

We found both initial models had a good fit based on our *a priori* definition: CFI = 0.97, TLI = 0.95, and SRMR = 0.02 and CFI = 0.99, TLI = 0.97, and SRMR = 0.02 for the physician and nurse measures respectfully. Given the large sample size, as expected the chi-square test for model fit was significant, p < 0.001 for both measures.

*Nested models*

Using confirmatory factor analysis we found a single factor model (both the physician and nurse 5-item compassion measures loading on a single latent variable) to have poor fit (CFI = 0.73, TLI = 0.65, SRMR = 0.13); however, we found a two factor model (physician and nurse 5-item compassion measures loading on separate latent variables) to have good fit (CFI = 0.97, TLI = 0.95, SRMR = 0.02). The likelihood ratio test comparing two nested models resulted in p < 0.0001. Thus, we reject the null hypothesis that the covariance between the two latent structures is 1 (i.e. the two-factor model has better fit).

*Mediation testing of nurse compassion and communication*

Nurse compassion was associated with nurse communication [β = 0.55 (95% CI 0.51 to 0.58)], and nurse communication partially mediated the association between nurse compassion and overall hospital rating [indirect effect, β = 1.27 (95% CI 1.06 to 1.47)]. Thus, nurse communication mediated 75% of the total association between nurse compassion and overall hospital rating. Physician compassion was not associated with nurse communication [β = 0.01 (95% CI -0.02 to 0.03)], nor did nurse communication mediate the association between physician compassion and overall hospital rating [β = 0.01 (95% CI -0.04 to 0.07)].

References

1. Centers for Medicare & Medicaid Services, HCAHPS Fact Sheet, (CAHPS® Hospital Survey) October 2019. available at <https://www.hcahpsonline.org>. Accessed September 21st, 2020.

2. Singer T, Klimecki OM**.** Empathy and compassion. Curr Biol. 2014;24(18):R875-R8.

3. Goetz JL, Keltner D, Simon-Thomas E**.** Compassion: an evolutionary analysis and empirical review. Psychol Bull. 2010;136(3):351-74.

4. Sinclair S, Norris JM, McConnell SJ, Chochinov HM, Hack TF, Hagen NA, et al. Compassion: a scoping review of the healthcare literature. BMC Palliat Care. 2016;15:6.

5. Roberts BW, Roberts MB, Yao J, Bosire J, Mazzarelli A, Trzeciak S**.** Development and Validation of a Tool to Measure Patient Assessment of Clinical Compassion. JAMA Netw Open. 2019;2(5):e193976.

6. Sabapathi P, Roberts MB, Fuller BM, Puskarich MA, Jones CW, Kilgannon JH, et al. Validation of a 5-item tool to measure patient assessment of clinician compassion in the emergency department. BMC Emerg Med. 2019;19(1):63.

7. Satorra A, Bentler PM**.** Corrections to test statistics and standard errors in covariance structure analysis. In Latent variables analysis: Applications for developmental research, ed. A. von Eye and C. C. Clogg. Thousand Oaks, CA, US Sage Publications, Inc.; 1994.

8. Kline RB**.** Principles and Practice of Structural Equation Modeling (2nd Edition ed.). New York: The Guilford Press; 2005.

9. Chen FF**.** Sensitivity of goodness of fit indexes to lack of measurement invariance. Structural equation modeling. 2007;14(3):464-504.

10. Cheung GW, Rensvold RB**.** Evaluating goodness-of-fit indexes for testing measurement invariance. Structural equation modeling. 2002;9(2):233-55.
